# Fertility-Sparing Surgery for Early-Stage Cervical Cancer

**DOI:** 10.1155/2012/936534

**Published:** 2012-07-08

**Authors:** Adelaide Fernanda Ribeiro Cubal, Joana Isabel Ferreira Carvalho, Maria Fernanda Martins Costa, Ana Paula Tavares Branco

**Affiliations:** Obstetrics and Gynecology Service, Gynecology Department, Centro Hospitalar Tâmega e Sousa, E.P.E., 4564-007 Penafiel, Portugal

## Abstract

Nowadays cervical cancer is diagnosed in many women who still want to have children. This led to the need to provide fertility-sparing treatments. The main goal is to maintain reproductive ability without decreasing overall and recurrence-free survival. In this article, we review data on procedures for fertility preservation, namely, vaginal and abdominal trachelectomy, less invasive surgery and neoadjuvant chemotherapy. For each one, oncological and obstetrical outcomes are analyzed. Comparing to traditionally offered radical hysterectomy, the overall oncologic safety is good, with promising obstetrical outcomes.

## 1. Introdution

Cervical cancer is the second most common cancer in women in developing countries and the seventh in developed countries [1]. It affects women of all ages, including those in their prime childbearing years. 

 More than 500.000 new invasive cervical cancers are estimated to be diagnosed worldwide every year. Because of the effective and widespread use of cervical carcinoma screening, many women will be diagnosed at a relatively young age and early stage [2]. The postponement of childbearing accompanied with the comparatively young age at which many women are diagnosed with cervical carcinoma has posed new challenges in the management of this disease—there is a strong demand for fertility-sparing surgery.

 Traditionally the recommended treatment for early cervical cancer is a radical hysterectomy (RH) with bilateral pelvic lymphadenectomy: removal of the uterus, cervix, radical resection of the parametrial tissue and upper vagina, and complete pelvic lymphadenectomy [3]. 

 Cervical cancer spreads laterally to the parametria, inferiorly to the vagina and rarely superiorly to the uterus [4–6]. This is why it is possible to maintain the fundus and adnexa in most small cancers confined to the cervix and thus maintain the possibility of future childbearing. 

 Parametrial removal in early cervical cancer remais important to rule out parametrial spread, which would be an indication for further therapy, to prevent local recurrence; and to obtain a clear margin of the cervical primary. 

 There are several types of fertility-saving procedures, which differ in terms of surgical approach and extent of paracervical resection. The most widely accepted is radical vaginal trachelectomy (RVT), but in the last years there are increasing reports of an abdominal approach to perform radical trachelectomy. There are also some less invasive procedures under investigation, such as large conization and simple trachelectomy. Neoadjuvant chemotherapy is being studied as a possibility to downstage larger tumors and allow for these fertility-sparing procedures. 

## 2. Selection Criteria

 The management of fertility sparing surgery must include a good selection of patients and complete information about them. They need to be informed about preoperative examinations, late complications, and especially the oncologic and obstetric outcomes related to the surgery as well as the alternative approaches [3]. There is no guarantee of fertility after a radical trachelectomy and the standard treatment for early-stage cervical carcinoma is still radical hysterectomy. So, detailed informed consent is essential [7–11].

 It is estimated that even with a careful patient selection for fertility-sparing surgery, 12–17% of the patients will have the procedure aborted due to nodal metastasis or positive endocervical margins [12]. 

 The main selection criterion is a strong desire to preserve fertility. Preservation of uterus in women who does not plan pregnancy is controversial [12, 13] as it is in women with previously impaired fertility. Assisted reproduction techniques are widely used and many women did not even tried to conceive before the diagnosis of cervical cancer. Hence, it is not possible to estimate reproductive potential before surgery accurately. 

 Most centres do not also specify an upper age limit for fertility-sparing surgery. Regarding their inherent risk of infertility based on age alone, some centres exclude patients from 40 or 45 years [2, 14–16].

 Tumor size is the most important risk factor for recurrence. It has been shown in many studies that tumors greater than 2 cm have a significant increase in the risk of recurrence [13, 17].

 Appropriate candidates for fertility-sparing surgery are patients with tumors of FIGO stage IA1 with lymphovascular space involvement, IA2 and IB1. Most centres include stage IB1 tumors of less than 2 cm only. 

 Tumor size may not completely exclude a candidate for surgery. For instance, a patient with an exophytic tumor with more than 2 cm but with little stromal invasion may still be a reasonable candidate for radical trachelectomy [44]. 

 Expert colposcopy is the standard examination before fertility-sparing surgery and is important in assessing the exocervical diameter and spread to the vagina [9, 15, 45].

 A second histopathological examination is important for determination of type, grade, tumor dimensions, depth of invasion, and lymphovascular space involvement. There is controversy as to whether adenocarcinoma or adenosquamous histology is related to a higher risk of recurrence, compared to squamous cell carcinomas. In the largest series published, which compared early-cervical cancers with different histological subtypes, it was found that adenocarcinomas and squamous cell carcinomas had similar outcomes with fertility-sparing surgery [46, 47]. Small-cell neuroendocrine carcinoma is not suitable for fertility-sparing surgery since the prognosis for this aggressive tumor is worse than for other types [13, 48, 49]. For this kind of tumor, usual treatment includes radical hysterectomy and chemotherapy. It is unknown if radical trachelectomy followed by chemotherapy would have the same outcomes [44]. 

 Lymphovascular space involvement is still the most commonly discussed risk factor. Although it is a negative prognostic factor for recurrence and nodal metastasis, its presence alone does not necessarily exclude the possibility of fertility-sparing surgery. There are reports in the literature of patients that underwent radical trachelectomy even with known lymphovascular space involvement, and only 5% of them were shown to have positive lymph nodes on specimen examination [50]. Patients should be informed of the risk of recurrence if lymphovascular space involvement is extensive [51].

 Magnetic resonance imaging (MRI) volumetry is another preoperative diagnostic method, and its information can be further amplified by the use of an endovaginal receiver coil [52] or by creating an artificial saline hydrocolpos [53]. It is important for determination of exact tumor size, amount of cervical stroma infiltration, and amount of healthy stroma (determination of tumor growth in anterioposterior, craniocaudal, and transverse directions). 

 Estimation of lesion size is further complicated when a patient has undergone conization prior to presentation for definite treatment [54]. 

 It has been shown that MRI has 100% positive and negative predictive value in assessing which patients are suitable for radical vaginal trachelectomy [55]. 

 Many clinicians have suggested that infiltration of less than half of the cervical stroma is the limit for a safe trachelectomy, because it is necessary to have a 1 cm free margin [15, 56, 57]. Some clinicians suggest margins of only 5–8 mm to be sufficient but this is still debatable [20]. 

 All forms of trachelectomy should save a good proportion of healthy stroma because the chance of successful pregnancy is higher. Preservation of the cervical stroma lowers the risk for cervical incompetence, ascending infection, premature rupture of membranes, and premature delivery [20]. 

 MRI can also assess tumor involvement of paracervical tissues. In the literature [5, 58], parametrial involvement in IB1 tumors ranges from 6 to 13%. Factors which potentially correlate with parametrial tumor spread at the time of radical hysterectomy include lymph node status, size of tumor, deep stromal invasion, stage, lymph vascular space invasion, grade, histology, and presence of residual tumor in the surgical specimen [58, 59]. 

 Patients with cervical cancer that has spread to the parametria require adjuvant chemoradiation and, therefore lose the benefit of the “fertility-sparing” aspect of the surgery [12, 54, 60]. In these patients, there may be an increased risk of complications. Unfortunately, most of the characteristics that increase the risk of spread (deep stromal invasion and vascular invasion) may not be determined reliably preoperatively [54]. 

 MRI and computer tomography (CT) scans are insufficient for evaluation of microscopic pelvic lymph node infiltration [61, 62]. A new generation of PET-CT and MRI, which use ultra-small iron particles, seems to be feasible for preoperative assessment of lymph nodes [63, 64]. Vaginal or rectal ultrasonography is also used in some centres, with good results [65]. 

 Usual clinical eligibility criteria for radical trachelectomy are listed on Table 1 [2].

## 3. Intraoperative Assessment

 During surgery, extrauterine spread to the lymph nodes should be assessed and an adequate margin of healthy stroma assured. Perioperative pathological examination should be performed. When extrauterine spread or infiltration of the cranial part of the specimen is found, it becomes necessary to perform a more radical surgery or to initiate chemoradiotherapy [20]. 

 Perioperative assessment of regional lymph nodes includes removal of the nodes from external, internal iliac, and obturator regions. They can be examined by repeated frozen sections but recently this assessment was replaced by detection of sentinel lymph node in many centres [13, 15, 17, 66]. The technique of sentinel lymph-node mapping may help localize aberrant nodal metastasis spread and identify micrometastases that have been missed with conventional histopathological processing [50]. In the presence of micrometastases (<2 mm) or isolated tumor cells not diagnosed until the final histopathology, adjuvant chemotherapy or radiotherapy should follow surgery, but there are no randomized studies about the ideal modality of treatment [20]. 

## 4. Radical Trachelectomy

Radical trachelectomy, the removal of the uterine cervix and adjacent tissues, was originally introduced in 1987 by Dr Daniel Dargent. He performed a laparoscopic pelvic lymphadenectomy and a VRT. In a short period of time, several centres presented studies regarding slightly modified VRT [45, 56, 67, 68] and also abdominal approaches [54, 69]. 

 The choice for abdominal or vaginal route as well as laparotomy or laparoscopic approach depends mainly on the surgeon's preference and level of expertise [3]. Details about the performance of these techniques are well described elsewhere [3, 19, 27–30, 32, 34, 60, 66, 68]. Robot-assisted laparoscopy is also rapidly increasing as a possibility in fertility-sparing surgery for early cervical cancer [70–73].

 The oncological safety of these procedures in the treatment of early-invasive cervical cancer is well established in many retrospective studies and is associated with an acceptable live birth rate [3, 9, 74].

 There has not been any randomized controlled trial comparing fertility-sparing radical trachelectomy to radical hysterectomy for the treatment of early cervical cancer. Such a trial is not feasible, since offering young women who desire fertility preservation a trial in which they would be randomized to a radical hysterectomy may be exceedingly difficult and unacceptable to these patients. Moreover, a formidable sample size would be needed to do meaningful statistical analysis. 

 Instead, there are some case-control studies [2, 22, 75] and a meta-analysis on five of these previous studies has been recently published, comparing 303 patients who underwent VRT with 892 who underwent RH. No significant differences were found between VRT and RH in 5-year survival rate, 5-year progression-free survival rate, intraoperative complications, and postoperative complications. There were fewer blood transfusions, less blood loss and shorter hospital stays in patients undergoing VRT. 

### 4.1. Surgical Complications

 Several studies compared the surgical morbidity of VRT with RH [47, 50, 76]. Overall VRT has equal or less morbidity than RH in terms of blood loss, surgery duration, analgesic requirements, and hospital stay [75, 76]. The main drawback of radical trachelectomy is the operative time associated with the procedure, which is in part caused by a longer learning curve. 

 Combined data showed an average intraoperative complication rate of 4% and postoperative complication rate of 12% [50]. 

 Bladder injury accounts for more than half of the complications; usually, it is easy to identify and repair, with no long-term sequelae. Vascular injuries are the second most common complications and occur mainly during lymphadenectomy or as a result of trocart insertion during laparoscopic procedures [50]. There are also reports of isolated cases of enterotomy, vaginal fornix laceration, and ureteral injury [27]. Lymphedema and lymphocyst formation are more common in RH [76]. However, there are two known cases of pelvic-obturator space lymphocysts infected by group B streptococcus associated with VRT [18]. 

 Typical complications reported after radical trachelectomy include dysmenorrhea (24%), dysplastic Pap smears (24%), metrorragia (17%), problems with cerclage sutures (14%), excessive vaginal discharge (14%), isthmic stenosis (10%), amenorrhea (7%), and occasional reports of deep dyspareunia [76]. 

### 4.2. Followup after Radical Trachelectomy

There are no universal guidelines as to the optimal followup after radical trachelectomy. 

 Most authors suggested visits every 3–6 months for the first two years, then every 6 months for three years. Typically, more than three-fourths of recurrences will occur within the first 2-3 years after the initial treatment but there have been reports of recurrence even 7 years after RVT. Thus, followups may be extended to every year after the first 5 years [56, 75, 77].

 Patients should be aware of symptoms of recurrence such as abdominal or pelvic pain, lymphedema, leg pain, vaginal bleeding or discharge, urinary symptoms, cough, and weight loss. They are present in 46–95% of the patients with recurrences [78, 79]. 

 Physical examination is widely accepted for surveillance and accounts for the highest detection rate when compared with cytologic evaluation and imaging modalities [80, 81]. It should include a complete assessment of areas that are susceptible to the human papilloma virus and a thorough speculum, bimanual, and rectovaginal examination. Along with symptoms, physical examination will detect most cases of recurrent cervical cancer [77]. 

 Although there is insufficient evidence, cytologic evaluation performance in retrospective studies showed low detection rates for recurrences and so it may not be mandatory. Nevertheless, most surveillance programs include cervical cytology, colposcopic examination, and eventually endocervical curettings. Follow-up cytology posttrachelectomy can have normal results interpreted as atypical so an experienced cytopathologist should be enrolled in interpreting results. It can also be important in detecting other lower genital tract malignancies [77, 82].

 Some clinicians perform routine MRI at 6, 12, and 18 months, while others do so only if clinically indicated [56, 75]. MRI should be read by radiologists familiar with the procedure since anatomic changes can be misinterpreted as recurrences [83]. Anyway, there is still insufficient data to support its routine use in asymptomatic patients. 

 PET scans have high sensitivity (86%) and specificity (87%) for detecting disease recurrence. Its use as a surveillance tool is also being studied with promising results [84, 85]. 

## 5. Less Radical Procedures 

 Approximately 65% of patients do not have any residual cancer in the trachelectomy specimen after a diagnostic cone [7, 9]. Additionally, the rate of parametrial involvement in patients with tumor size ≤2 cm, negative pelvic nodes, and depth of invasion ≤10 mm is only 0,6%, so it might be safe not to resect parametrial tissue in these patients [58, 86–88]. This raises the question as to whether less radical surgery provides similar effectiveness to RVT. 

Recently, some authors proposed less radical procedures for “low-risk” patients (tumor size <2 cm, low risk histology, absence of lymph vascular space invasion) [42]. 

Usual protocols perform pelvic lymphadenectomy first, and if there are no positive nodes (or if sentinel node is negative), a large conization or simple trachelectomy is performed after. Simple trachelectomy consists of amputation of the cervix approximately 7–10 mm above the lesion and then removal of the endocervical channel by use of loop electrosurgical excision. This technique keeps the risk of stenosis to a minimum [89]. 

## 6. Neoadjuvant Chemotherapy and Fertility Sparing Surgery

 In women affected by larger cervical lesions (>2 cm tumor size), there is a higher risk of recurrence [90]. Some authors suggested the use of neoadjuvant chemotherapy prior to surgery in these patients [42], providing a more conservative endocervical tissue resection, diminishing the risk of central recurrence, and potentially improving obstetrical results. 

 Concerning the deleterious effects of chemotherapy on ovarian function, this treatment should be offered to women who normally have a good ovarian reserve, since alkylating agents such as ifosfamide and cisplatin can be detrimental to ovarian follicles.

 Different chemotherapy protocols include (1) cisplatin 75 mg/m^2^ plus ifosfamide 2 g/m^2^ every 10 days. (2) cisplatin 75 mg/m^2^ plus doxorubucin 35 mg/m^2^ every 10 days; (3) TIP: paclitacel 175 mg/m^2^ plus cisplatin 75mg/m^2^ plus ifosfamide 5 g/m^2^; (4) TEP: paclitacel 175 mg/m^2^ plus cisplatin 75 mg/m^2^ plus epirubicin 80 g/m^2^, every 21 days; TEP is usually used in adenocarcinomas. In the future, less gonadotoxic regimens should be evaluated [21]. 

 However, downstaging tumors larger than 2 cm by neoadjuvant chemotherapy is still an experimental procedure and will need multicentre cooperation to verify its oncological safety.

## 7. Oncological and Pregnancy Outcomes

The critical concern when treating patients with early-stage cervical cancer is whether conservative surgery is as effective as the standard radical hysterectomy. 

 In some instances, patients will be recommended to receive additional treatment due to the presence of positive lymph nodes, close or positive upper margins of the removed cervix, or unusual histological subtype as neuroendocrine carcinoma. Therapy can consist of radical hysterectomy or radiation, with or without chemotherapy; this depends on the center protocol and the timing of diagnosis—intraoperative versus postoperative [50]. Even after an appropriate patient selection, it is estimated that around 10% of the patients would require these additional treatments and thus will lose the fertility-sparing characteristic of the procedure. 

 There are some reports of patients who refused adjuvant therapy when indicated. Three women with nodal micrometastasis refused adjuvant treatment and none recurred. Four women with positive nodes on final pathology refused radiation therapy and did only chemotherapy and none recurred. Two patients with margins inferior to 5 mm on the superior cervical canal on final pathology also refused adjuvant therapy and none recurred [17, 90]. 

 Yet, in other series, there are reports of one patient that had close margins and recurred in the uterine fundus after 3 months and another patient with invasive cancer after 10 months [17]. 

 Until now, there have been many reports on oncological outcomes of RVT, which are described in Table 2.

 In a total of 849 women, only 83 (9,8%) for whom a VRT was planned could not have their fertility preserved, mostly because of positive nodes. 

 Recurrence rate was 3,9%. Excluding one article which does not specify tumor size, a comparison of recurrences in tumors less than 2 cm in size—2,6%, with recurrences in bigger tumors—23,9%, shows that VRT might be a risky procedure for tumors larger than 2 cm. 

 In women who underwent VRT, mortality rate was 3,1%. 

 There have been 436 pregnancies reported after fertility-sparing VRT, which resulted in 279 deliveries—see Table 3. Excluding ongoing pregnancies, delivery rate was 67,2%. The rate of first trimester miscarriage was 22,4%, which is similar to that of the general population. The rate of second trimester miscarriage was 10,3%—twice higher than that of the general population, mainly because of ascending infections and premature rupture of membranes. Premature delivery also had a higher rate and occurred in 74 of 279 deliveries—26,6%. Various authors suggested routine administration of antibiotics between 14–16 weeks, antepartum management with prophylactic antibiotics, bimonthly screening for infections, bed rest, steroids therapy, and even serial measurements of cervical length [91, 92]. It appears that none of these approaches are evidenced-based and all of them require further investigation, although it is a consensus that these pregnancies should be followed up as high-risk pregnancies [75].

There have also been reports on oncological outcomes of ART but data is less extensive—see Table 4. Of the 251 cases reported, fertility-sparing surgery was not possible in 12% of women because of lymph node involvement. 

 Oncological outcomes of ART were good and similar to those of VRT as there were only 7 recurrences reported (3,4%). 

 In a comparison of recurrences in tumors less than 2 cm in size—1,2% with recurrences in bigger tumors—12,5% shows that ART, as VRT, is also a risky procedure for tumors larger than 2 cm. 

 The obstetrical outcomes reported with ART—see Table 5—have been less than with VRT, as a result of less experience with this procedure, and also because of recommendations of some clinicians to wait 2 years prior to conception [32].

 In all 221 women that have undergone ART, it was found that only 35 women achieved pregnancy, which is a dramatically lower rate of pregnancies than that found with VRT [8, 20]. The rate of pregnancy loss (23,3%) was similar to that in VRT, and preterm labor was slightly bigger (44,4%). 

 It is generally believed that the difference in pregnancy rates between vaginal and abdominal radical trachelectomies is due to the fact that in RVT the blood supply from the main uterine arteries is not affected, while the uterine artery is usually transected at its origin in ART [93].

 However, some facts reported in other studies contradict this theory: healthy pregnancies at term have developed even with the uterus being perfused relying only on the ovarian vessels [94] and there is also an ART performed in a patient who was 15-week pregnant, and despite the need to completely transect the left uterine vasculature, the pregnancy reached term without evidence of any anomaly, including fetal growth restriction [95]. So, further data with long-term followup need to be gained to determine whether preserving the uterine artery is an important factor in improving pregnancy outcomes [73]. 

Preliminary findings for less invasive surgeries such as large conization or simple trachelectomy after pelvic lymphadenectomy (or sentinel node identification) are comparable to those achieved with abdominal or vaginal radical trachelectomies—see Table 6. In patients with negative lymph nodes and tumors less than 2 cm, results are promissing and comparable with the results of VRT and ART [20]. Prospective multicentric studies will be needed to confirm their oncological safety. 

Of the 46 surgeries performed, there was only one recurrence reported; in this case adjuvant treatment with chemotherapy was performed, and there was no evidence of disease until now (5-year followup) [15, 89]. 

 Half of the women become pregnant after surgery and there were reported 30 pregnancies, and 19 deliveries—see Table 7. These studies, although in a small scale, show that less-invasive procedures have good results and have the potentiality of performing even better than radical trachelectomy in selected patients. 

Oncological and pregnancy outcomes after neoadjuvant chemotherapy and fertility-sparing surgery were reported in few series—Tables 8 and 9. 

 In a total of 41 fertility-spared women, there were only 3 recurrences registered, one of which occurred in the ovary and the patient died soon after. All recurrences occured in patients in whom the surgery performed was less radical than radical trachelectomy [40]. 

There were 23 pregnancies in 18 of the 41 women who undergone neoadjuvant chemotherapy before surgery—Table 9. There were one first-trimester loss, five preterm deliveries and 15 full-term babies. 

 Analysis on pregnancy outcomes for all different approaches revealed that ART performed worse than all the others and that less radical procedures had significantly better results as it would be expected [20].

 The extent of the removed cervix, the technique of re-anastomosis, and the formation of the neocervix are factors than will affect future fertility, because of shortening of the cervix length, diminished cervical mucus, and stenosis of the residual cervix. All techniques try to save as much cervix as possible, leaving at least 1 cm of cervical stroma. Approximately 15% of patients develop cervical stenosis after RVT [96]; most are asymptomatic, but some develop menstrual disorders or hematometra, requiring dilatation of the cervical ostium to resolve. Another important factors that affect fertility are the higher risk of abdominal surgical adhesions, subclinical salpingitis and disruption of the uterus and tube innervation after pelvic lymphadenectomy, and parametrial resection [50, 75]. 

 It was estimated that infertility rate after trachelectomy is between 25–30% [75]. 

 In patients with difficulties conceiving after trachelectomy, a complete infertility workup should be done, and patients may require assisted reproductive techniques as any other case. In 75% of the cases, a cervical factor appears to be the cause for the infertility [75]. 

 There is no consensus as to the timing of pregnancy after RT. Some suggest a 6 months to 1-year followup period before attempting pregnancy [9, 44], but others do not establish any period [56]. 

## 8. Conclusion

The management of early-stage cervical carcinoma in young women who desire future fertility remains a challenge to gynecologic oncologists. 

 Tumor size, presence of positive nodes, lymphovascular space involvement, deep stromal invasion, and unfavorable histology are the most important risk factors for recurrence and should be carefully evaluated preoperatively. 

Nowadays, radical vaginal trachelectomy is a well-established safe procedure on early cervical cancer with large experience to date. It has good oncological and obstetrical outcomes with low morbidity and mortality, especially in tumors less than 2 cm in size. 

 Experience with abdominal open or laparoscopic approach is increasing, and it is now possible to select patients for less radical fertility-sparing procedures such as large cone biopsy or simple trachelectomy. Neoadjuvant chemotherapy before fertility-sparing surgery is an innovative approach, which can extend the possibility of a conservative treatment to many young women affected by larger cervical lesions. 

 New data from these techniques is currently being studied, and in the future more options will be safely available for early cervical cancer such as the use of robotic surgery in large institutions, which will result in surgeries performed safer, better, faster, and at a lower cost. 

## Figures and Tables

**Table 1:** Eligibility criteria for radical trachelectomy.

FIGO stage IA1 with lymphovascular space involvement, IA2 and IB1	
Desire for future fertility	
Age ≤ 40–45 years	
Confirmed invasive carcinoma—squamous, adenocarcinoma, or adenosquamous	
No previous documentation of infertility (+/−)	
No evidence of pelvic lymph node metastasis and/or other distant metastasis	
Patient being a candidate for surgery	
4–6 weeks postconization with adequate resolution of acute inflammation	

**Table 2:** Characteristics and oncological outcome of RVT.

Authors	Planned surgeries	Fertility preserved	Positive nodes	LVSI	Histology			Recurrence		Deaths
					SCC	AC	O	≤2 cm	>2 cm	
Shepherd et al. [7, 8]	158	138	7	49	103	51	4	3^∗∗^		4
Sonoda et al. [18]	43	36	2	NA	24	16	3	1/36	0/0	1
Pahisa et al. [14]	15	13	0	1	9	6	0	1/11	1/2	1
Chen et al. [19]	16	16	0	1	14	2	0	0/9	0/7	0
Hertel et al. [17]	108	106	2	38	74	33	1	3/105	1/1	2
Dargent et al. [2, 20]	135	118	9	43	90^∗^	25^∗^	3^∗^	1/91	6/27	5
Plante et al. [21]	140	125	9	32	69^∗^	48^∗^	8^∗^	3/111	3/14	2
Covens et al. [7, 22]	93	91	2	31	40	50	3	5/83	1/8	4
Burnett et al. [12, 23]	21	18	1	6	12	9	0	0	0	0
Schlaerth et al. [24]	12	10	0	1	4^∗^	5^∗^	1^∗^	0/10	0/0	0
Mathevet et al. [13]	108	95	8	23	76^∗^	18^∗^	1^∗^	0/85	4/8	3

Total	849	766	40	225	559	264	24	33		24

^∗^Only after VRT; ^∗∗^data not available for the number of recurrences > and ≤2 cm; NA: not available data.

**Table 3:** Pregnancy outcomes of VRT.

Authors	Fertility preserved	Pregnant women	Conceptions	Abortions		Deliveries		
				1st T	2nd T	Preterm	Term	On going^∗^
Shepherd et al. [7, 8]	138	NA	88	22	12	10	37	7
Sonoda et al. [18]	36	11	11	3	0	0	4	4
Pahisa et al. [14]	13	3	3	0	0	0	1	2
Chen et al. [19]	16	5	5	0	2	1	1	1
Hertel et al. [17]	106	18	18	3 (2VIP)	0	8	4	3
Dargent et al. [2, 20]	118	33	56	14	8	5	29	0
Plante et al. [21]	125	58	106	25 (4VIP)	4 (1VIP)	19	58	0
Covens et al. [7, 22]	91	18	24	3	3	6	12	0
Burnett et al. [12, 23]	18	3	3	0	1	1	1	0
Schlaerth et al. [24]	10	4	4	0	2	1	1	0
Mathevet et al. [13]	95	33	56	14	8	5	29	0
Danska-Biazinska et al. [25]	14	2	2	1	0	0	1	0
Speiser et al. [26]	212	50	60	8 (2VIP) (1EP)	3	18	27	4

Total	992	238	436	93	43	74	205	21

^∗^Ongoing pregnancies at the time of publication of each study; VIP: voluntary interruption of pregnancy; EP: ectopic pregnancy; NA: not available data.

**Table 4:** Characteristics and oncological outcome of ART.

Authors	Planned surgeries	Fertility preserved	Positive nodes	LVSI	Histology			Reccurrence		Deaths
					SCC	AC	O	≤2 cm	>2 cm	
Abu Rustum et al. [27, 28]	22	15	6	9	9	13	0	NA	NA	0
Pareja et al. [29]	15	14	1	5	11	4	0	0/14	0/0	0
Nishio et al. [30]	71	61	15	31	58^∗^	2^∗^	1^∗^	1/48	5/13	NA
Cibula et al. [31]	24	17	4	2	14	10	0	1/14	0/3	NA
Ungará et al. [32]	33	30	2	8	26^∗^	1^∗^	3^∗^	0/21	0/9	0
Olawaiye et al. [33]	10	10	0	1	3	7	0	0/9	0/1	0
Wan et al. [34]	2	2	0	0	2	0	0	0/2	0/0	0
Yao et al. [35]	10	10	0	NA	8	2	0	0/10	0/0	0
Li et al. [36]	64	62	2	4	50^∗^	8^∗^	4^∗^	0/48	0/14	0

Total	251	221	30	59	181	47	8	2/166	5/40	0

^∗^Only after ART; NA: not available data.

**Table 5:** Pregnancy outcomes of ART.

Authors	Fertility preserved	Pregnant women	Conceptions	Abortions		Deliveries		
				1st T	2nd T	Preterm	Term	On going^∗^
Abu Rustum et al. [27, 28]	15	2	2	1	0	0	0	1
Pareja et al. [29]	14	3	3	0	0	1	2	0
Nishio et al. [30]	61	4	4	0	0	2	2	0
Cibula et al. [31]	17	6	6	1	0	2	3	0
Ungará et al. [32]	30	13	13	4	0	1	5	3
Olawaiye et al. [33]	10	3	3	1	0	1	1	0
Wan et al. [34]	2	0	0	0	0	0	0	0
Yao et al. [35]	10	2	2	0	0	1	1	0
Li et al. [36]	62	2	2	0	0	0	1	1

Total	221	35	35	7	0	12	15	5

^∗^Ongoing pregnancies at the time of publication of each study.

**Table 6:** Characteristics and oncological outcome for less radical procedures.

Authors	Planned surgeries	Fertility preserved	Positive nodes	LVSI	Histology			Reccurrence	Deaths
					SCC	AC	O		
Rob et al. [15]	40	32	6	17	32	7	1	1	0
Landoni et al. [16]	11	11	0	NA	5	6	0	0	0
Bisseling et al. [37]	3	3	0	NA	0	3	0	0	0

Total	54	46	6	17	37	16	1	1	0

NA: not available data.

**Table 7:** Pregnancy outcomes for less radical procedures.

Authors	Fertility preserved	Pregnant women	Conceptions	Abortions		Deliveries		
				1st T	2nd T	Preterm	Term	On going
Rob et al. [15]	32	17	23	5	3	12		3
Landoni et al. [16]	11	3	3	0	0	0	3	0
Bisseling et al. [37]	3	3	4	0	0	4		0

Total	46	23	30	5	3	19		3

**Table 8:** Characteristics and oncological outcomes for fertility-sparing surgery after neoadjuvant chemotherapy.

Authors	Planned surgeries	Fertility preserved	Positive nodes	LVSI	Histology			Reccurrence	Deaths
					SCC	AC	O		
Maneo et al. [38]	21	16	2	1	9	12	0	0	0
Kobayashi et al. [39]	1	1	0	0	1	0	0	0	0
Plante et al. [21]	3	3	0	0	3	0	0	0	0
Robova et al. [40]	15	12	0	9	9	3	0	3	1
Palaia et al. [41]	1	1	0	0	1	0	0	0	0
Marchiole et al. [42]	8	7	1	NA	6	2	0	0	0
Gottschalk et al. [43]	1	1	0	0	0	1	0	0	0

Total	50	41	3	10	29	18	0	3	1

NA: not available data.

**Table 9:** Pregnancy outcomes for fertility-sparing surgery after neoadjuvant chemotherapy.

Authors	Fertility preserved	Pregnant women	Conceptions	Abortions		Deliveries		
				1st T	2nd T	Preterm	Term	On going
Maneo et al. [38]	16	6	10	1	0	2	7	0
Kobayashi et al. [39]	1	1	1	0	0	1	0	0
Plante et al. [21]	3	2	3	0	0	1	2	0
Robova et al. [40]	12	7	7	0	0	1	5	1
Palaia et al. [41]	1	0	0	0	0	0	0	0
Marchiole et al. [42]	7	1	1	0	0	0	0	1
Gottschalk et al. [43]	1	1	1	0	0	0	1	0

Total	41	18	23	1	0	5	15	2
